# Tongue Hematoma With Necrosis

**DOI:** 10.7759/cureus.12741

**Published:** 2021-01-16

**Authors:** Jerina Nogueira, Ana Rita Parente, André Mendes, Isabel Lavadinho

**Affiliations:** 1 Internal Medicine Department, Unidade Local de Saúde do Norte Alentejano - Hospital Dr. José Maria Grande, Portalegre, PRT

**Keywords:** warfarin, miconazole, clarithromycin, tongue hematoma, tongue necrosis, adverse side effect

## Abstract

Spontaneous tongue hematoma is a known rare adverse side effect associated with warfarin therapy. There is a long list of drug-to-drug interactions with warfarin that may contribute to a rise in international normalized ratio (INR) levels, increasing the risk of bleeding.

We present a case of an elderly female patient who presented with oral dysphagia and spontaneous oral cavity bleeding while on warfarin therapy. She was found to have tongue hematoma and necrosis. A week prior she started treatment with topical miconazole for oral candidiasis and a few days later topical clarithromycin was added. Treatment given was mainly supportive with intravenous vitamin K, fresh frozen plasma, and aminocaproic acid. Full recovery was achieved.

It is our intention to raise awareness of a rare adverse side effect related to warfarin therapy that may have been precipitated with the use of medications known to contribute to INR elevation.

As learning points, we emphasize close monitoring of INR levels when using known drug-to-drug interactions with warfarin and also consider replacing warfarin for a direct oral anticoagulant if no contraindication is present.

## Introduction

Warfarin is a well-known oral anticoagulant and its major adverse side effect is bleeding (1-10%). This risk may be minimized with monthly control of the international normalized ratio (INR) as a guidance to dose adjustment. However, close monitoring is seldom sufficient in minimizing this risk given that many other factors may contribute to reducing the dose-response relationship, e.g. other medications, diet high in vitamin K, alcohol, infections, and patient susceptibility. Moreover, necrosis of the skin or other tissue is considered a rare adverse reaction occurring in less than one percent of patients on warfarin therapy [1]. 

A large number of drugs interact with warfarin, through different pharmacokinetic and pharmacodynamic mechanisms and, presently, the list continues to increase [2].

Elderly patients are commonly polymedicated and drug interactions are frequent. Clinicians go through a daily struggle to avoid these events which can impose a serious health problem.

Clarithromycin has for a long time been known to increase patient’s INR levels when administered to patients on regular use of warfarin [3]. Miconazole oral gel, used to treat oral candidiasis, is also known to negatively interact with warfarin by elevating INR levels and increasing the risk of bleeding events [4-6]. Unfortunately, many clinicians are still unaware of this interaction [6].

There are several case reports [7-14] regarding spontaneous tongue hematoma associated with warfarin usage with elevated INR levels on admission. It is a rare complication related to warfarin therapy, but can lead to potentially life-threatening complications requiring orotracheal intubation due to significant oropharyngeal edema and quite possibly surgical intervention. Some cases were managed by administering vitamin K intravenously along with fresh frozen plasma to lower INR levels while simultaneously suspending warfarin intake and maintaining close monitorization.

With this report, we plan to raise awareness of the rare adverse side effect of tongue hematoma related to warfarin usage which, in this case, could have been precipitated by clarithromycin and miconazole combination. We also intend to share how we managed this situation in the hope of aiding other clinicians that may encounter a similar clinical case.

## Case presentation

A woman in her early 80s with a one-week history of progressive sore throat and dysphagia arrived at the emergency department (ED) due to a spontaneous oral cavity bleeding. No traumatic event was recognized. 

Approximately one week prior to the presentation, she was diagnosed with oral candidiasis. On the day of the presentation, she was on day six of topical miconazole 20 mg/g three times daily and day four of clarithromycin oral suspension 40 mg/ml 5ml twice daily. Clarithromycin was later added to miconazole due to the worsening of the patient’s symptoms raising suspicion of bacterial co-infection.

Her past medical history was significant for pulmonary embolism of unknown etiology, hypertension, and rheumatoid arthritis. Her home medications included warfarin (dosage according to INR with monthly monitoring), losartan 100 mg/day, and methotrexate 15 mg/week. She had no known drug allergies.

During her clinical evaluation, the patient was found alert and hemodynamically stable with a peripheral capillary oxygen saturation of 99% without oxygen supplement, and no signs of breathing difficulty. She was, however, unable to speak.

**Figure 1:**
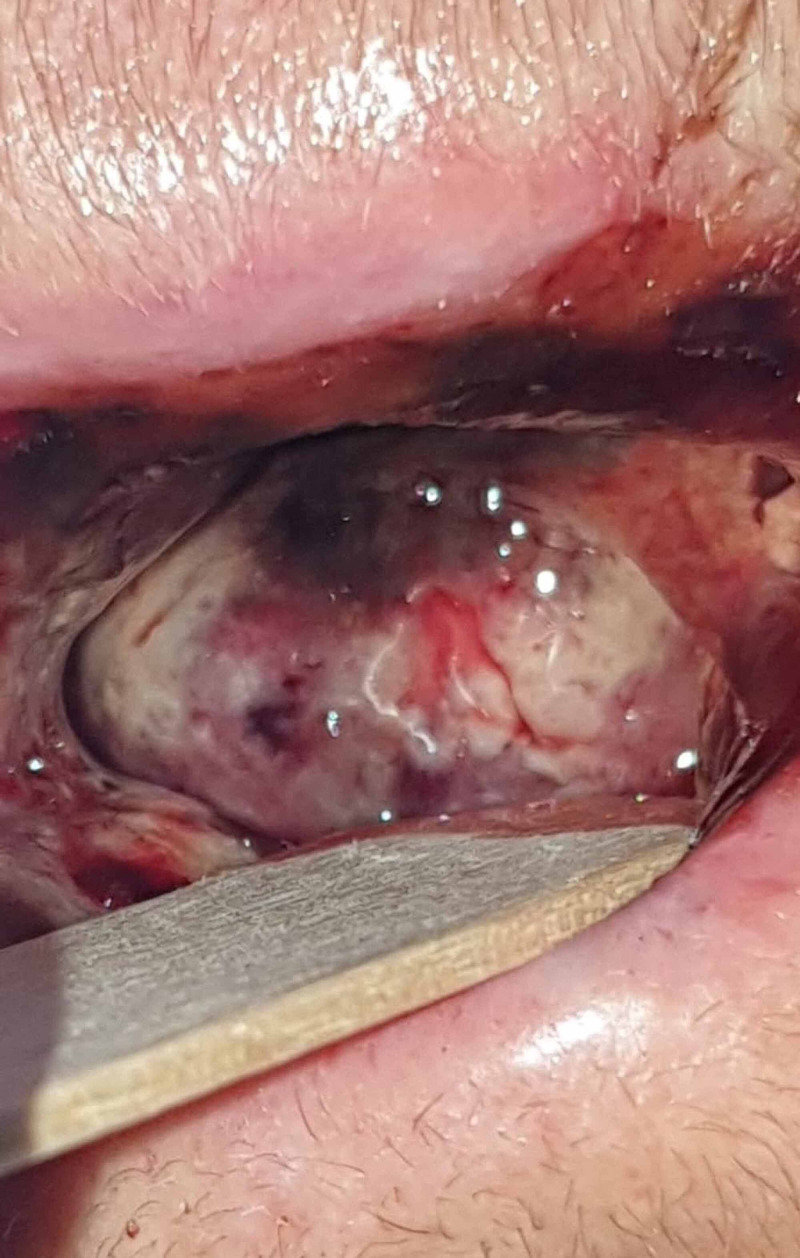
Tongue hematoma and necrosis at admission

On examination of the oral cavity, she presented red-blackish discoloration of the anterior portion of the tongue with non-viable fibrous yellow tissue. The upper and lower inner lip portions were also affected (Figure 1).

Sublingual hematoma could not be ruled out, but there was no evidence of swelling on palpation of the anterior triangle and no sign of ecchymosis, although tongue mobility was reduced. There was active moderate bleeding, but the source could not be pinpointed. Due to the severe hematoma, no other observation of the oral cavity was possible at the time.

There were no signs of respiratory distress, presenting with normal breathing sounds upon chest auscultation. She denied any presence of pain, except when trying to open her mouth.

Otorhinolaryngology is not available in our hospital so a multidisciplinary team of Internal Medicine and General Surgery professionals gathered to discuss patient evaluation and treatment.

Laboratory findings showed: low hemoglobin 8.9 g/dl; low hematocrit 25.7%; normal mean corpuscular volume (MCV) 91.1 fl; normal mean corpuscular hemoglobin (MCH) 31.6 pg; low leukocyte count 670 uL (neutropenia 250 uL and lymphocytopenia 340 uL); low platelet count 53,000 uL; very high INR >12; and high c-reactive protein 99.4 mg/L.

A maxillofacial and neck computed axial tomography scan was performed which ruled out sublingual hematoma and airway obstruction. Tongue biopsy was initially considered, but given the high risk of uncontrolled bleeding this invasive intervention was postponed. 

Stevens-Johnson Syndrome (SJS) was thought to be a possible diagnosis, but the patient did not have any fever, malaise, myalgia, or arthralgias. Even though the oral mucosa was severely affected, no other skin/mucosal lesions were found, more specifically, ocular involvement. The lab tests did detect anemia, lymphopenia, and neutropenia which are common in SJS, but no other common laboratory findings were detected such as electrolyte imbalance, increased blood urea nitrogen, or elevated serum aminotransferase levels. Also, the drugs she was medicated with were not the most commonly associated with this syndrome. This being, overall findings were not very consistent with this diagnosis [15].

The patient was urgently treated with intravenous vitamin K 10 mg, two fresh frozen plasma units, and aminocaproic acid 5 g, and was closely monitored. Besides warfarin, methotrexate was also suspended due to the suspected myelosuppression found in this patient, which is a reported possible adverse side effect of methotrexate even in low dosages [16]. Transferring the patient to a tertiary hospital was considered by the team if she did not respond to initial treatment.

Within 24 hours, she showed a decrease in hemoglobin (6.2 g/dl) for which she received a transfusion of two units of compatible blood type. The bleeding stopped completely within approximately 36 hours of admission and INR levels started to decrease. Visible exudate and white plaques were found on the oral mucosa suggesting co-infection (bacterial vs fungal). Considering the myelosuppression and evidence of a significant rise of c-reactive protein to 332.9 mg/L in the lab analysis, piperacillin-tazobactam and fluconazole were empirically added.

In the following days, the patient presented favourable improvement. Initially, she had some difficulty swallowing since she was unable to properly use her tongue, but two days after admission she began to eat small amounts of cold liquefied food with no signs of dysphagia.

**Figure 2:**
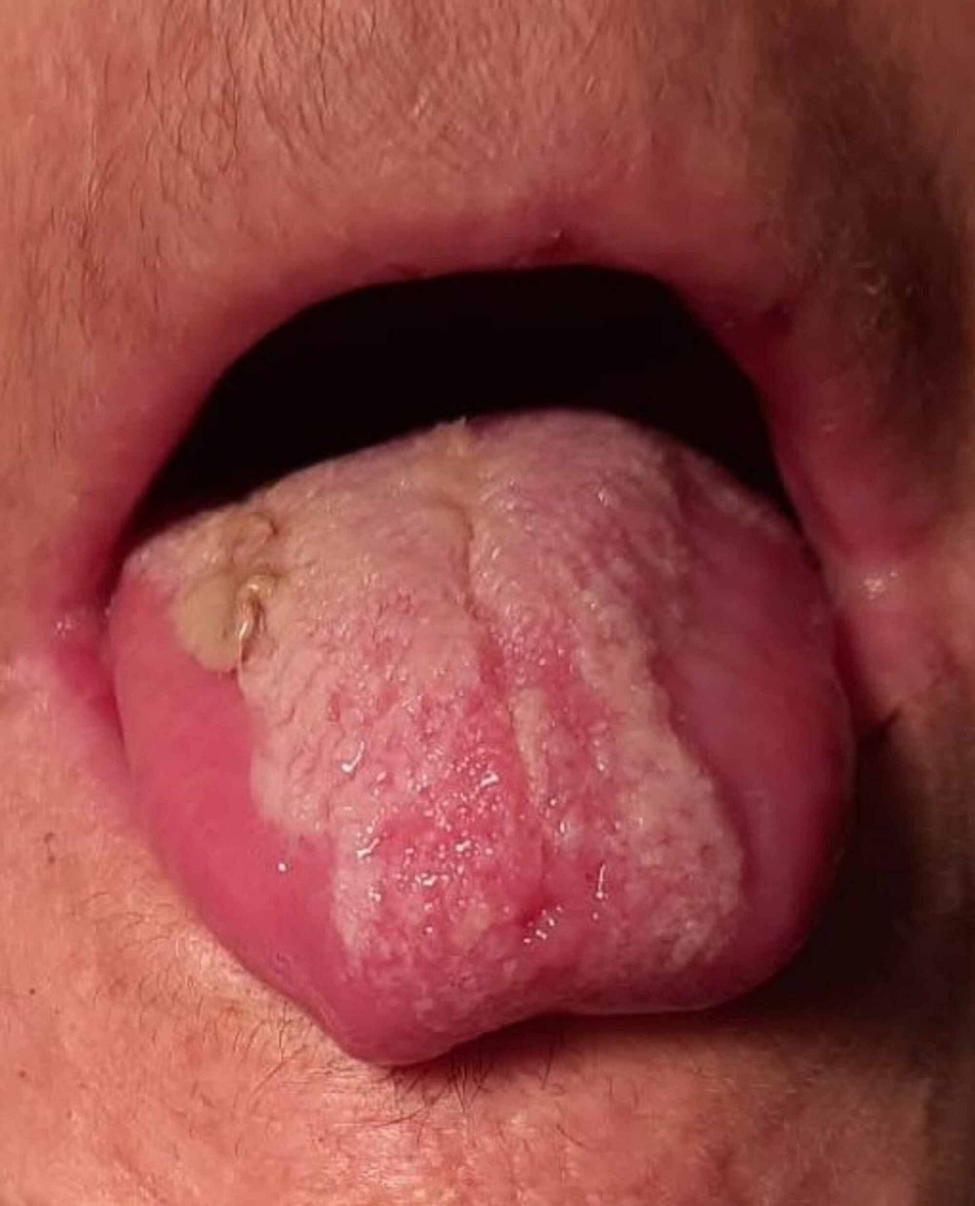
Tongue hematoma and necrosis resolution seven days after admission

By the seventh day of hospitalization, she completed her antibiotics and was speaking without any difficulty. She was able to eat and drink normally with no pain associated. On examination of the oral cavity, we found normal mobility of her tongue, no signs of hematoma, edema or infection, pink in colour and a fibrin plaque suggestive of a near-complete healing process (Figure 2).

Laboratory findings showed that leukopenia was resolved (leukocyte count 6410 uL, neutrophil count 4000 uL, and lymphocyte count 1150 uL). Thrombocytopenia was also resolved (platelet count 409,000 uL) and c-reactive protein levels were now 43.5 mg/L in a consistent decreasing trend. INR levels were 1.29.

Given her past medical history of pulmonary embolism, we cautiously decided to maintain anticoagulant therapy, but decided to avoid re-starting warfarin after verifying no contraindications for introduction of a direct oral anticoagulant. We chose apixaban, a direct inhibitor of factor Xa.

Although tongue biopsy was once again considered, since she presented notable improvement it was decided that this procedure was no longer beneficial. The patient was safely discharged one week after admission and oriented to an ambulatory follow-up.

## Discussion

In Internal Medicine, we commonly evaluate and follow polymedicated elderly patients with multiple comorbidities. This is a daily challenge, since life expectancy continues to increase and new medical treatments continually arise adding to the number of medications on the patient's list. This being, it is of the utmost importance to avoid drug-to-drug interactions in order to reduce possible adverse events that can be life-threatening.

Spontaneous tongue hematoma is a rare adverse side effect associated with warfarin therapy. In the present clinical case, this risk would have been exacerbated with the use of clarithromycin and miconazole since both are known to negatively interact with warfarin by increasing INR levels. Discontinuing medication and combining supportive therapy were the main treatments offered to this patient, presenting a very successful outcome.

SJS could be a possible differential diagnosis, but the rapid improvement seen in this patient and the absence of other common features do not point in this direction. It may be questionable if oral candida infection played a leading role in the development of tongue hematoma but, to the best of our knowledge there are no reports of such specific relation. 

With so many documented warfarin drug interactions, it can be particularly challenging to identify all of them and sometimes we are left with no other option than to knowingly use these medications when there is no better alternative.

## Conclusions

Supportive care and the reversal of anticoagulation with vitamin K and fresh frozen plasma appear to be a safe choice when addressing spontaneous tongue hematoma associated with warfarin. Orotracheal/nasotracheal intubation can quite suddenly be needed to secure the airway. With this in mind, we advise close monitoring and supportive care. Monitor INR levels more closely when there is a need for combining medications known or suspected to interact negatively with warfarin and cause INR levels to increase. Whenever possible, consider replacing warfarin with a direct oral anticoagulant since they are associated with fewer interactions.
